# **“**We don't get much of a voice about anything”: perspectives on photovoice among people who inject drugs

**DOI:** 10.1186/s12954-019-0334-2

**Published:** 2019-11-27

**Authors:** Mari-Lynn Drainoni, Ellen Childs, Katie B. Biello, Dea L. Biancarelli, Alberto Edeza, Peter Salhaney, Matthew J. Mimiaga, Angela R. Bazzi

**Affiliations:** 1 Section of Infectious Diseases, Department of Medicine, Boston University School of Medicine, Boston, MA USA; 2 Evans Center for Implementation and Improvement Sciences, Boston University School of Medicine, Boston, MA USA; 3 Department of Health Law, Policy & Management, Boston University School of Public Health, Boston, MA USA; 4 Center for Healthcare Organization and Implementation Research, Edith Nourse Rogers Memorial Veterans Hospital, Bedford, MA USA; 5 Center for Health Equity Research, Brown University, Providence, RI USA; 6 Department of Behavioral & Social Sciences, Brown University School of Public Health, Providence, RI USA; 7 Department of Epidemiology, Brown University School of Public Health, Providence, RI USA; 8 The Fenway Institute, Fenway Health, Boston, MA USA; 9 Department of Psychiatry & Human Behavior, Brown University Alpert Medical School, Providence, RI USA; 10 Department of Community Health Sciences, Boston University School of Public Health, Boston, MA USA

**Keywords:** Substance abuse, Intravenous, Vulnerable populations, Qualitative research, Participatory action research, Photography, Photovoice, Health services research, Ethics

## Abstract

**Background:**

In the context of the current US opioid crisis, people who inject drugs (PWID) are increasingly researched, but their ability to tell their own stories may be limited. Photovoice is a participatory action research method that allows participants to use photography to directly depict their experiences.

**Methods:**

We conducted interviews with PWID (*n* = 33) as part of a qualitative study on the health needs of PWID in the USA to explore interest and acceptability of photovoice as a potential research method and way to share their voices.

**Results:**

Participants identified facilitators and barriers to participating in a future photovoice project. Facilitators included a chance to depict one’s unique experience, help others in need by sharing one’s own story, and photography being a more “comfortable” way to tell their stories than traditional research methods. Barriers included safety concerns, embarrassment, and ability to retain cameras. Participants also identified areas of sensitivity related to documenting drug use.

**Conclusions:**

While we found broad acceptability of photovoice, barriers would need to be addressed and additional training and support for research staff and potential participants related to the ethics of public photography and engaging PWID in photovoice research would be required.

## Introduction

Enhancing research participation among marginalized populations is critical to understanding their needs and perspectives. However, specific barriers to participation may exist in research studies using traditional designs and methods. Due to stigma and legal concerns, people who inject drugs (PWID) may be hesitant to participate in research studies. Commonly used research methods to understand PWID’s experiences and health needs include surveys, interviews, focus groups, and participant observation [1, 2]. However, even in qualitative studies that allow individuals speak for themselves, individuals’ experiences are mediated through the lens of the researcher. In light of increasing overdose deaths from opioid and polysubstance use and injection {Zibbell, 2018 #3588;Zibbell, 2015 #3587} [3, 4] and recent outbreaks of human immunodeficiency virus (HIV) and hepatitis C infections [5, 6] in the USA, it is critical that the voices of PWID be heard more directly. Understanding the world through their eyes can help create and improve harm reduction and health and social services for this vulnerable population.

Photovoice is a participatory action research method that has been used successfully with PWID. It provides participants with opportunities to document and reflect on their community and individual needs, converse about important topics by sharing photographs, and reach policymakers and other community stakeholders with insights into particular areas of concern [7–11]. Photovoice has been widely used with marginalized populations [12–17] including people who use drugs [18–21] to provide insights into their unique experiences. In studies of individuals who use drugs, visual methods have helped to explore and illuminate the complicating factors of homelessness, poverty, lack of access to healthcare [18–20], and stigma and discrimination [20]. However, photovoice projects with people who use drugs have focused less on strategies to support access to healthcare services, which is an increasingly important avenue for research in the context of the US opioid crisis. Importantly, most of the published photovoice research with people who use drugs have been conducted outside of the USA [12–15], and we have not identified any published research studies using photovoice with US PWID.

The existing literature on using photovoice and other visual methodologies with marginalized populations identifies important barriers to acceptability and ethical concerns related to issues of privacy of photographed subjects [16–18], protecting the best interests of the participants [14, 18], and responding to stigma against and negative responses to marginalized identities or illegal activities that may be depicted [14, 15, 19]. To engage PWID in photovoice research in an ethical way, it is important to incorporate these previously identified ethical considerations in study design decisions, weighing the benefits of hearing the unique “voice” of PWID with potential ethical and practical challenges.

Despite increasing research and programmatic attention to the US opioid crisis, the voices of PWID remain obscured. To develop innovative, practical responses to the health harms of drug use and injection, it will be vitally important to elicit PWID’s perspectives on barriers to accessing services and potential solutions. As part of a qualitative study on the health needs of PWID in the US Northeast, a region heavily impacted by opioid use and injection, overdose deaths, and HIV and HCV transmission among PWID, we explored the acceptability of photovoice research methods. In this paper, we examine PWID’s perspectives on the potential facilitators and barriers to participating in photovoice research, highlighting the key role that the PWID “voice” should play in ongoing research on the opioid crisis.

## Methods

### Study design and sample

We drew from a qualitative study exploring PWID’s perspectives on pre-exposure prophylaxis (PrEP) for HIV prevention among 33 PWID in Boston, MA, and Providence, RI [20]. PWID were recruited in partnership with local community-based organizations (CBOs) including syringe-exchange programs and drop-in HIV/HCV testing centers. Trained study personnel screened interested individuals for eligibility, which included being ≥ 18 years old and self-reporting past-month injection drug use and HIV-uninfected status. Purposive sampling helped maximize diversity in participants’ demographics and HIV risk behaviors [21, 22]. Participants provided verbal informed consent prior to interviews. The Boston University Medical Center Institutional Review Board approved all study protocols.

The sample included approximately equal numbers of PWID in the two cities (Providence: *n* = 17; Boston: *n* = 16; total *n* = 33). Overall, median age was 36 years (interquartile range 31.5–48). Most identified their race as White (67%) or Black (21%); 24% of participants identified as Hispanic. Slightly over half identified as male (55%) and heterosexual (64%). Most were unemployed (70%) and had a high school diploma or equivalent (39%) or less (27%). Participants described active injection drug use, with over a third (36%), injecting 2–3 times daily, and an additional 15%, injecting ≥ 4 times daily.

### Data collection

From October 2016 to October 2017, trained qualitative interviewers conducted confidential interviews in private offices or other spaces within CBOs. Interviewers administered brief demographic and behavioral questionnaires and then used semi-structured interview guides with open-ended questions on PrEP as well as broader health and healthcare challenges. In developing the interview guide, the study team was cognizant of the stigma and other healthcare barriers experienced by PWID [20, 23, 24] and importance of taking a community-engaged approach to research. We thus included questions about engaging in a photovoice research project. We first explained how some research projects give people cameras to document their lives and daily activities and asked participants about their opinions regarding participating in such research. All interviews were audio-recorded with participants’ permission.

### Data analysis

Interview recordings were professionally transcribed verbatim for analysis. We reviewed transcripts for accuracy. Analysis followed an inductive approach drawing from the procedures of grounded theory and the constant comparative method [25–27]. To create the codebook, we used a collaborative process [28, 29] involving six research team members who initially each independently reviewed three transcript excerpts to develop potential codes. This coding team then met to discuss their findings and develop an initial codebook with preliminary definitions. The team independently coded another set of three transcripts and met again to revise the codebook by refining existing codes and definitions to allow for a better “fit” of new data and add newly developed codes. After establishing consensus on the final codebook through additional rounds of this process, three team members independently coded all remaining transcripts using NVivo (QSR International Pty Ltd., version 11, 2017) and met weekly to review consistency in code application, resolve coding discrepancies, and discuss emergent themes. For this analysis, we identified themes relating to the acceptability of using photography in research with the goal of understanding if and why participants might participate in photovoice research. Key findings are described below and exemplified using representative quotes.

## Results

### Overview of qualitative themes

Twenty six out of 33 participants indicated that they would be interested in using cameras to depict their lives and help tell their stories. Of the remaining seven study participants, four said they absolutely would not want to participate in a photovoice project and three were unsure. Of those participants who were interested, they explained the reasons why, what would facilitate their participation, and potential barriers to participation. There were three strong facilitators of participating in a future photovoice research project: (1) participants believed it would allow them to give “voice” to their unique experience, especially in a visual way, (2) participants thought it could help other PWID and “give back” to others in need, and (3) it would enable them to participate in research that could be more “comfortable” than traditional surveys or interviews. Despite primarily positive perspectives, even those who said they would be interested identified three potential barriers to participating in a future photovoice project: (1) safety risks related to photographing others, (2) embarrassment about their situations related to their drug use and current lives, and (3) concern about their ability to hold onto study cameras. Participants also explained what they would and would not be willing to photograph. These common facilitators, barriers, and specific subjects of high sensitivity are described below.

### Facilitators to photovoice participation

#### A chance to voice one’s unique experience

Participants who were interested in participating in a photovoice project spoke powerfully about the value of giving a “voice” to their unique experience. As one participant said, “*I think some people might be interested in [photovoice] because we don't get much of a voice about anything”* (B09). Relatedly, participants were also interested in the visual nature of photovoice, believing that visually depicting their everyday experiences for others would help give them a voice, *“like a recording of your lifestyle*” (B14). Similarly, another participant summarized how depicting his life would not only give voice but provide purpose and meaning, stating:

*“That would be neat. It’s like…a reality show, like hey, today I had this to do and do that…That would give a person something else to do. And talk, to feel like they're talking to someone, instead of not talking to anyone at all. So, that can probably help someone out, like, okay I have to do this and… whoever sees this is like, oh, so this is where the person went wrong, you know, or they’re voicing it out so by them saying it out loud to something or someone, might prevent them from doing what they actually are tempted to do”* (P06).

A participant who believed himself to be different from other PWID expressed a strong desire to share his unique experience:

*“Yeah, I would do [photovoice] because I think my experience is a little unique compared to a lot of [others]. It's not as common for many reasons… a lot that we didn't even talk about…like here, a lot of people[’s] stories are very similar. Mine’s kind of really different… I grew up [and] I was a white boy…in an area where the white boys were only known to come and buy drugs…”* (B08).

Another participant perceived value in documenting his life for the world, stating, “*If you put a camera on me, that would be the best show ever…The shit that I go through every day is incredible…It’s like out of a movie”* (P08).

#### Help others in need and give back to the community

Participants also explained that they felt giving voice to their experience through pictures could be a good way to “give back” and help others in need, as one participant explained:

*“It would be like getting some information out there, and the more that anybody can get help, the better, you know…, Any little bit can help. Maybe it will be something I hear or something like that that'll just click”* (P12).

Another participant described how a photovoice project could help increase awareness of the risks associated with drug use:

“Maybe it will [make] people aware…of the dangers of drugs, you know, sex drugs, drugs with money, sex-with-drugs, thing[s] that they’re really living…Maybe it can wake them up [to] realize once you get something you can’t erase it. Once you get HIV or AIDS, you cannot erase that. Once you get herpes, you cannot erase that” (B12)*.*

Despite some privacy concerns, one participant said that as long as his participation could be anonymous, he would participate in photovoice to help others: *“if it was something [to] educate more people about it [drug use]”* (P13). Another simply said, *“I would be [interested] because I know it could help somebody”* (P04).

#### A way to participate in research that felt comfortable

Finally, participants viewed photovoice as an opportunity to participate in research that would be more “comfortable” than responding to surveys or qualitative interviews. One participant said: *“I would definitely do that. And a lot of times I don’t really know what to say, so pictures are different…I would like that better, I’d like that better….You don’t have to talk”* (B14). Another participant echoed that sentiment saying, “*the [photo]voice thing would be another excellent idea because that way maybe somebody would be shy and don't want to talk, [they] could hear somebody else speak about it and see their daily usage and the picture and thing like that. And they say, ‘Wow. This guy is doing it, let me do it too’*” (B12).

### Barriers to photovoice participation

#### Risks related to taking photos of others

Although most participants expressed interest and willingness to participate in a photovoice study, they raised some concerns about others misinterpreting their photo-taking as an extension of police surveillance and would potentially retaliate. As one participant explained,

“To get a bunch of other people is going to be a lot of trouble, because, they running their mouth, they going to say you snitching, they get busted, they say probably you set them up. All kinds of bullshit could come out of that...Some people can think that you taking pictures to give to the police…or you work as an informer. And a lot of us, they can end up in big bullshit trouble” (P07)*.*

Concerns related to feeling monitored and being suspicious of who would see photographs were prominent. Even recognizing that the purpose of the photovoice research would not involve disclosure to police, one participant voiced concern: “*You never know who could see that camera”* (B02). Another participant talked about how it could put him in danger of being harmed:

*“That would very difficult because the people that I’m around, that are a lot of cocaine users...either they would A) swipe if from me; or B) they would start thinking that I was documenting for the police. And it would not go very well for me personally…and could cause bodily harm to me”* (P10).

#### Embarrassment about their situations related to drug use and current lives

Despite many participants saying they wanted to use their stories to help others, some expressed hesitation because they were embarrassed about putting their lives on display for public viewing. As one participant explained, *“Nah, I can’t…I’d be too nervous...Embarrassed. Plus, I don’t like people to know what I’m going through”* (P09). Another participant spoke of living two different lives, one at work and one at home, and not wanting people to see the difference:

*“I wouldn't personally [participate], only because of my life. I work every day, I'm in a medical type setting. But if I had a different way of living, then yeah. If I didn't care as much, or if I [didn’t] have to hide so much, then yeah it would be something I'd do”* (P02).

Another participant voiced a preference for personal privacy, saying, *“I don't know, it's just... even now when I do anything like [use drugs] I do it in private, so, it would just feel weird”* (P12).

#### Concerns about retaining cameras

A common concern raised by study participants was related to how their daily lives would affect their ability to retain study cameras or phones to use in photovoice. Because many participants reported being homeless or unstably housed, they were worried about cameras being stolen:

*“I mean you'd probably get a couple of cameras gone in the process, but it all depends on how long…I mean if it's just a one day thing or a weeklong thing... 'Cause there's nothing you can do if someone takes your stuff and you're sleeping, you know?”* (B01).

Other participants described difficulty keeping track of their possessions, especially cellphones:

*“I think some people would have trouble. Just ‘cause a lot of people, like me, I can’t…I’m horrible, I’ll lose a phone in two seconds. It’s always ‘cause it falls out of my pocket. That’s why I lose ‘em, ‘cause I wear the sweatshirts with the big pocket in the front, and then when I sit down, your sweatshirt kinda goes up like this”* (B04).

### Specific subjects of high sensitivity

Although the large majority of participants said they would participate in photovoice research, individuals expressed some varying opinions about what they would feel comfortable documenting via photography. While some individuals felt comfortable documenting anything in order to depict their experiences and help others, others thought they would be more judicious in their selections of photographic subjects in order to protect their safety and that of their peers. One participant reported high levels of willingness to document his own drug use, similar to what he had seen in documentaries on television:

*“I've seen it before…Documentaries on TV where they go into somebody's house, they use drugs…And the people that are using drugs, they let them record them while setting it up, prepping it up, and shooting in, and getting high…I've actually done it before on my own, know what I'm saying? I'd be all high or whatever. I'd set my phone up and set to record myself getting high. Know what I'm saying? Even going through the steps where I'd talk to the cameras, say, ‘See, right now I'm doing this. I'm setting it up… that’s the cooker and I put the heroin or cocaine, whatever, into the cooker. That's step one, step two.’ And I'd go through the steps, you know”* (P11).

Other participants were more hesitant to document illegal or stigmatized activities. One participant explained that he would not record buying drugs because, “*I would get in a lot of trouble*”. The same participant described ways to mitigate potential ethical concerns by asking permission before taking photos, saying, *“I mean, some of the same people you would have to ask for consent for people to be on the camera*” (P08). Another participant reported knowing where to go to take photos, but voiced concerns about taking photos of people when they are high and “out of it,”

*“I wouldn't be opposed to it, it all depends on… I wouldn't want to be out there trying to snap pictures of other people and get myself into a mix, but, like I said, I know all the spots out here where people go to use, I know, on a daily basis I watch five people who are just like out of it, you know, like, I wouldn't be opposed to it but I would have to know like what they wanted”* (B01).

While discussing concerns related to taking pictures of people, one participant suggested providing clear details about who and what to photograph in the study description and instructions, including, *“Just things you’re doing…Yeah, I think that’ll be a big point to bring up. And then it might change people’s mind [to participate] a little more”* (B04).

Among the participants who said they would absolutely not participate, reasons were primarily related to general fears of monitoring, including concerns that their information would be shared with the police. Relatedly, even among individuals who would participate, participants expressed concerns about taking pictures of others and the potential violence that could result. Finally, one person spoke about the belief that photovoice would be more appropriate for people who were in treatment and not currently injecting drugs.

## Discussion

Despite the stigma associated with injection drug use [23, 30], most participants in this study indicated a willingness to potentially participate in photovoice research because they believed it could help document their unique experiences, bring voice to their experience, and possibly help others in similar situations. Some participants believed that participating in photovoice research would be easier, more comfortable, and would better enable them to voice to their perspectives than participating in studies using more traditional research methods such as surveys or interviews. Our findings are consistent with literature showing that individuals in socially marginalized groups view photovoice as a helpful method for directly depicting their experiences [11, 31]. Also consistent with prior work [7], {Budig, 2018 #3606}the participants in our sample believed that this highly participatory approach would allow them to better tell their stories, share their experiences, and document their daily realities.

Beyond being useful as a data collection method, photovoice as an intervention has been found to reduce self-stigma and improve coping. Photovoice interventions have empowered participants through increased personal growth and promoted recovery in persons with serious mental illness [32]. Photovoice may also help combat societal stigma HIV/AIDS [31, 33, 34]. The participants in our study identified desiring privacy around their drug use as a potential barrier to participating in a future photovoice project, possibly due to fear of exposure, criminalization, or stigma. Photovoice should be considered as an intervention to help PWID cope with internalized stigma while raising societal awareness and humanizing the lived experiences of PWID to combat societal stigma against substance use.

Despite embarrassment about their situations and concern about legal and other potential risks from participation in a hypothetical photovoice study, many participants in this study expressed a desire to document their experience as a way to help others. Importantly, some participants wanted to share their stories to provide a cautionary tale to others, perceiving that depicting their experiences through photographs could help *“wake [others] up”* and show “*where the person went wrong”.* While these prevention-oriented perspectives are important, more general depictions of the lived experiences of individuals who are currently using drugs could help raise awareness of substance use and the challenges that people who use drugs experience in their daily lives. Indeed, photovoice has been used to cultivate visual evidence on under-investigated issues and topics that are often misunderstood including disability, accessibility [35, 36], housing, and homelessness [37]. An important component of many photovoice projects is dissemination through an exhibit of the photographs taken, which can help educate policymakers and the broader community on these topics [9, 38, 39]. By raising societal awareness of substance use and addiction, this specific type of activity could help reduce societal stigma against injection drug use.

Considerable literature discusses the ethics of photovoice and other visual methodologies [40–43]. Central ethical topics and concerns for visual methodologies that also arose in our study include confidentiality, consent (both for participants and photographed subjects), and safety [14, 16, 18, 19, 44, 45]. While the parent study from which we drew data was not designed to investigate specific ethical issues within photovoice research with this population, our participants nevertheless raised many of these same ethical concerns. Although most participants said that they would be willing to participate in a photovoice project, some did acknowledge that there were things they would be hesitant to photograph including active injection drug use or drug purchasing. Researchers considering photovoice with PWID should thoroughly engage with this literature on ethics within visual methodologies [40–43]. In conducting a photovoice project with PWID, it would be vital to provide specific training related to confidentiality, personal safety, and consent [46, 47] including considerations for avoiding photographing individuals who may not wish to be photographed [8, 34, 47] and guidelines about how to photograph illicit activities [34]. Any potential use of photovoice with PWID includes addressing the ethical issues related to full consent and perceived safety and risks for vulnerable populations that have been well documented elsewhere [14–19].

Several practical challenges may also exist in photovoice research with PWID. Participants were concerned about potential loss or theft of cameras, which would inhibit their ability to fully participate and share photos. However, technology could support the immediate submission of photos to the research team to save and print for participants, mitigating part of the effect of losing cameras. What is important about this but has not been previously addressed in the literature is how the issue of being unable to hold on to belongings points to about the daily challenges life instability can bring. It would also be important to structure the project in a way that can be flexible and adapt to the needs and lifestyles of individuals living with addiction who may be unstably housed and only able to participate sporadically or inconsistently.

Findings from this qualitative study should be considered with some limitations in mind. First, although we purposively sampled diverse PWID, we conducted our study in two Northeastern cities and our findings may not generalize to other US regions. Second, the original study was not solely focused on photovoice, and the questions on it were asked toward the end of interviews. We may have missed opportunities to probe to more fully explore interest and willingness to engage in photovoice research. Third, our participants were asked about their willingness to participate in hypothetical research photovoice project in the future, so their perceptions may not reflect everything that would happen if they were actually participating in a real project.

Despite these limitations, our findings support the use of photovoice as a potentially powerful research methodology for understanding the perspectives and experiences of a stigmatized and marginalized population. Our study finds that PWID were open and interested in potentially participating in a photovoice project, with many participants describing advantages of photovoice over participation in traditional research. Participants viewed photovoice as a way to help others better understand their experiences, and to overcome the perception that PWID lack a voice to tell their own story. If the ethical challenges are fully addressed and the logistical issues can be overcome, photovoice is a potentially acceptable method of conducting research in partnership with this population, and may meet a critical need in the context of the size and tragedy of the US opioid crisis.

## Data Availability

The datasets generated and/or analyzed during the current study are not publicly available given the sensitive nature of the interview topics.

## Abbreviations

CBOs: Community-based organizations
HIV: Human immunodeficiency virus
PrEP: Pre-exposure prophylaxis
PWID: People who inject drugs
